# Effects on patient-reported outcomes of “Screening of Distress and Referral Need” implemented in Dutch oncology practice

**DOI:** 10.1007/s00520-019-05140-1

**Published:** 2019-11-28

**Authors:** Floor M. van Nuenen, Stacey M. Donofrio, Marrit A. Tuinman, Harry B. M. van de Wiel, Josette E. H. M. Hoekstra-Weebers

**Affiliations:** 1 Wenckebach Institute, University of Groningen, University Medical Center Groningen, Groningen, The Netherlands; 2 Netherlands Comprehensive Cancer Organisation, Utrecht, The Netherlands; 3 Department of Psychology, University of Groningen, Groningen, The Netherlands; 4 Department of Health Psychology, University of Groningen, University Medical Center Groningen, Groningen, The Netherlands

**Keywords:** Effect study, Distress screening, Patient-reported outcomes, DT&PL

## Abstract

**Purpose:**

This study investigated the effect of the “Screening for Distress and Referral Need” (SDRN) process (completing a screening instrument; patient-caregiver discussion about the patient’s responses, regardless of distress level, and possible referral to specialized care), implemented in Dutch oncology practice on patient-reported outcomes (PROs).

**Methods:**

A non-randomized time-sequential study was conducted to compare two cohorts. Cohort 1 respondents (C1) were recruited before and cohort 2 respondents (C2) after SDRN implementation in nine Dutch hospitals. Participants completed the EORTC-QLQ-C30, HADS, Patient Satisfaction Questionnaire-III, and the Distress Thermometer and Problem List (DT&PL). Descriptive analyses and univariate tests were conducted.

**Results:**

C2 respondents (*N* = 422, response = 54%) had significantly lower mean scores on the practical (*t* = 2.3; *p* = 0.02), social (*t* = 2.3; *p* = 0.03), and emotional PL domains (*t* = 2.9; *p* = 0.004) compared with C1 (*N* = 518, response = 53%). No significant differences were found on quality of life, anxiety, depression, satisfaction with care, distress level, the spiritual and physical PL domains, or on referral wish.

**Conclusions:**

After implementation of SDRN, patients report significantly fewer psychosocial (practical, social, and emotional) problems on the DT/PL but responses on the other patient-reported outcomes were comparable. These results add to the mixed evidence on the beneficial effect of distress screening. More and better focused research is needed.

## Introduction

Distress screening guidelines have been developed to ensure that medical specialists and nurses gain insight into distress and cancer-related problems of cancer patients regularly and that timely and justified referral of distressed patients to additional professional care takes place [1–4]. The aim is to prevent worsening of and/or treat cancer-related distress and problems and to maintain or improve quality of life (QoL). However, the results reported in the literature on the effect of distress screening on patient-reported outcomes (PRO) are inconsistent. Results vary from significant benefits on primary outcomes, such as (aspects of) QoL, depression, anxiety, and/or symptom burden (e.g., [5–8]), and secondary outcomes such as patient-care provider communication and quality of care, to no significant effects on such outcomes (e.g., [9, 10]). No studies reported a negative effect of distress screening.

The varying results may be due firstly to the differences in study design. Some studies used a randomized controlled design while others used a cross-sectional cohort pre-post implementation sequential design or an observational design.

Secondly, the instruments used vary between studies. Some studies used a non-cancer-related questionnaire like the Hospital Anxiety and Depression Scale [11, 12], while others used a cancer-specific questionnaire like the European Organization for Research and Treatment of Cancer - Quality of Life Questionnaire (EORTC-QLQ) [13] or the Functional Assessment of Cancer Therapy-General (FACT-G) [14]. Some recent studies used the instrument used for screening as outcome measure [6–8]. To our knowledge, only one study [8] has reported results on the Distress Thermometer and the Problem List (DT&PL) as outcome measure although this instrument is used for screening worldwide [15, 16].

Thirdly, differences in results of the effect of distress screening could be explained by differences in the distress screening procedure used. The method used to screen patients reportedly varies from only informing responsible medical specialists that a certain patient has a score above a cut-off to a more comprehensive process including triage. More studies using a more comprehensive distress screening process including triage found positive effects for patients than studies that examined a simple process consisting of patients completing a screening instrument that was made available to a healthcare provider who then decided what to do with it. The extent to which healthcare providers actually used patient information on a screening instrument during consultations or if they discussed responses on a completed screening instrument with all or some patients, such as only those having a score above a cut-off, remains unclear in most of the studies published [4].

In the Netherlands, the Comprehensive Cancer Organisation Netherlands, location Groningen (IKNL-G), implemented a process of “Screening for Distress and Referral Need” (SDRN) in routine clinical practice of general hospitals and one university medical center in the IKNL-G’s catchment area together with professionals in these hospitals. Previously, in none of these hospitals did SDRN or another form of distress screening take place. The SDRN process involves (1) regular completion of the Dutch version of the DT&PL by patients during (curative or palliative) treatment and follow-up; (2) discussion of DT&PL responses between care provider and patient, regardless of the DT-score being below or above the cut-off; (3) referral to psychosocial and/or allied healthcare providers based on the DT&PL responses and the discussion. The Dutch guideline advises that this comprehensive distress screening process takes place during a patient’s hospital visit with either the oncologist or the nurse at least every 3 months during treatment and follow-up [4]. Additionally, providing information about the goal of SDRN and the DT&PL and about the expertise of psychosocial and allied healthcare professionals to whom the patient could be referred to was considered essential [17, 18].

The aim of the current 2-cohort pre-post implementation study is to examine the effect of SDRN implemented in daily practice in hospitals on patients’ cancer-related distress, problems, and referral wish, on patients’ QoL, anxiety, and depression, and on their satisfaction with care. We hypothesize that the effects are positive.

## Method

### Design

This study employed a comparative 2-cohort pre-post implementation sequential design.

### Participants

Eligible patients were newly diagnosed patients, and patients in treatment or follow-up who visited a medical, surgical, gynecological, or urological outpatient clinic of one of the nine hospitals willing to implement SDRN and to participate in the study. Other inclusion criteria were that patients had to be aware of their cancer diagnosis and treatment plan; 18 years or older; and cognitively and physically able to answer questionnaires in the Dutch language. Patients with a psychiatric diagnosis were excluded.

### Procedure

Cohort 1: The first cohort of cancer patients (C1) was recruited between January 2007 and December 2010 in nine hospitals in the Netherlands. These hospitals had decided to implement the SDRN process; C1 was recruited pre-implementation. Hospitals were given a pre-determined number of packages consisting of written information about the study, the questionnaire, an informed consent form, and a pre-franked return envelope. The number of packages varied between hospitals from 30 to 200 depending on the number of cancer patients newly diagnosed per year in each particular hospital (Dutch Cancer Registry, IKNL). Nurses and medical specialists informed all eligible patients about the goal of the study during a regular outpatient clinic medical visit and gave them the package. Informing patients and handing out packages took between 2 and 4 weeks. Patients were asked to complete the questionnaire at home and send it in the supplied pre-franked envelop to IKNL-G. For reasons of anonymity, no information was given to IKNL-G by the hospitals about the patients approached to participate, and thus how many patients accepted the package.

Once the pre-determined number of packages was handed out, implementation of SDRN started according to a pre-developed roadmap including procedure and materials in the participating hospitals. In short, IKNL-G representatives approached hospitals and informed stakeholders in oncology care about the importance of SDRN, supported implementation, provided centralized project management, and organized mono- and multidisciplinary meetings for professionals of the hospitals to share experiences with implementation and execution of SDRN in clinical practice. Hospitals were urged to appoint a team leader and to form a multidisciplinary team [17].

Cohort 2: After SDRN had been implemented for at least a year according to a hospital, the second cohort (C2) was recruited from the first nine hospitals that met these criteria. Procedure and inclusion criteria were equal to cohort 1, with one exception. The (written) information about the study that C2 participants received was slightly different. C1 respondents were informed that a quality improvement project involving screening for distress was going to be implemented in the hospital and that they were invited to participate in a study assessing their QoL and satisfaction with care received before implementation. C2 respondents were informed that SDRN had been implemented and that we invited them for a study assessing their QoL and satisfaction with care received after implementation. Both groups were informed about the goal of SDRN. There was no overlap in patients between cohorts.

The medical ethical committee of the University Medical Centre Groningen decided that no approval was needed for the study. The study was performed according to the ethics committees of the participating hospitals and the Helsinki Declaration.

### Instruments

Participants completed questions on socio-demographic (age; gender; highest education completed (low (elementary or low vocational school), middle (secondary or middle vocational education), high (high vocational or university)); having children (yes or no, living at home or out of the house); and illness-related characteristics (date of diagnosis, cancer type, type of treatment, and treatment phase (watchful waiting, under active treatment, or follow-up)). Types of treatment were dichotomized into non-intensive (surgery only, radiotherapy only, and watchful waiting) and intensive treatment (all other treatment modalities). Based on the type of cancer and treatment, a medical oncologist placed patients in a curative or a palliative treatment intent group.

The Dutch Distress Thermometer and Problem List (DT&PL) [15] was used as instrument in the SDRN process and as patient-reported outcome measure. Patients indicated their cancer-related level of distress during the last week on the DT on an 11-point Likert scale from 0 (not distressed) to 10 (extremely distressed). The cut-off score found was 5 (sensitivity = 85%; specificity = 67%). On the 47-item PL (*α* = .90), patients could indicate whether they experienced problems in the following domains: practical (7 items (*α* = .60)); social (3 items (*α* = .75)); emotional (10 items (*α* = .80)); spiritual (2 items (*α* = .64)); and physical (25 items (*α* = .74)). The scoring scale on a problem item ranges from 0 (no, not a problem) to 1 (yes, very mildly troubled) to 10 (yes, extremely much troubled). Five PL domain summary scores were computed by taking the total score of the items in each domain. Possible ranges depended on the number of items in each domain and varied from 0–20 to 0–250. Lastly, respondents indicated if they wanted a referral for the problems they experienced (no, maybe, or yes).

The European Organization for Research and Treatment of Cancer, Quality of Life Questionnaire-C30 (EORTC-QLQ-C30) [13], a frequently used (nationally and internationally), valid and reliable, self-report, 30-item questionnaire was used to assess cancer patients’ quality of life. The EORTC-QLQ-C30 consists of the five functioning subscales: physical (5 items (*α* = .71)), emotional (4 items (*α* = .80)), role (2 items (*α* = .52)), social (2 items (α = .77)), and cognitive functioning (2 items (*α* = .73)); and the global quality of life subscale (2 items (*α* = .89)). Subscale scores were computed and transformed to a range from 0 to 100 according to the manual. Higher scores on the functioning and global QoL subscales indicate a higher QoL.

The 14-item Hospital Anxiety and Depression Scale (HADS) [11, 12] is a widely used tool to measure mild mood disorders in non-psychiatric patients. It consists of the two 7-item subscales anxiety (*α* = .83) and depression (*α* = .82). Patients are asked to indicate how they feel during the last 2 weeks on a 4-point scale ranging from 0 to 3. Subscale scores range from 0 to 21 and the total score from 0 to 42. Higher scores indicate higher symptom severity.

The 42-item Patient Satisfaction Questionnaire-III (PATSAT) [19] assesses patient satisfaction with oncological care on four aspects: technical quality (10 items (*α* = .74)), interpersonal behavior (14 items (*α* = .89)), accessibility (12 items (*α* = .76)), and general satisfaction (6 items (*α* = .92)). Total satisfaction represents the score of all questions. Patients could indicate their agreement on a 5-point scale ranging from agree completely (1) to disagree completely (5). After summation of items, subscale scores are transformed into a range from 0 to 100. Higher scores indicate higher satisfaction.

### Analysis

Descriptive analyses were conducted for the socio-demographic and illness-related characteristics, and for the patient-reported outcome measures. *T* tests (for continuous variables) and chi-square tests (categorical variables) were used to compare C1 with C2 on patient’ characteristics and the outcome measures. Effect sizes (Cohen’s *d*) were computed when groups were found to differ significantly to examine if these differences were clinically relevant. Cohen’s *d* < 0.2 were considered negligible, those between 0.2 and 0.49 small, those between 0.50 and 0.79 moderate, and those > 0.80 large [20]. At the request of pastoral workers, two hospitals did not use the two sub-items in the spiritual domain for C2 respondents, thus reducing the number of respondents for this domain with *N* = 85. Analyses were performed with SPSS version 23.

## Results

A total of 518 respondents participated in C1 (response = 53%) and 442 respondents in C2 (response = 54%). Response rates were comparable between hospitals. Table 1 shows patient’ characteristics and comparisons between the two cohorts. Cohorts were equal with respect to age, education level, having children, children living at home, treatment type, intensity, and phase. Groups differed significantly in gender (*X*^2^ = 8.8; *p* = 0.003), type of cancer (*X*^2^ = 32.1; *p* ≤ 0.001), time since diagnosis (*t* = 5.6; *p* ≤ 0.001), and treatment intent (*X*^2^ = 7.1; *p* = 0.008). In comparison to C1, more C2 respondents were female, and more were diagnosed with breast cancer while fewer with digestive cancer. Time since diagnosis was significantly shorter in C2, and fewer C2 respondents were treated with a palliative intent.

**Table 1:** Socio-demographic and illness-related characteristics of the respondents in cohorts 1 (pre-implementation) and 2 (post-implementation), and comparison between cohorts

Characteristics	C1 (*N* = 518)	C2 (*N* = 442)	Test value	*p* value
Age (mean ± SD) (range)	59.8 ± 11.5 (27.5–89.0)	59.3 ± 10.5 (29.9–87.5)	*t* = 0.74	0.46
Missing (*N*)	7	5		
Gender (*N*(%))			*X*^2^ = 8.8	0.003
Men	138 (27)	82 (19)		
Women	380 (73)	360 (81)		
Education (*N*(%))			*X*^2^ = 1.3	0.52
Low	131 (26)	103 (24)		
Middle	246 (48)	203 (47)		
High	131 (26)	125 (29)		
Missing	10	11		
Having children (*N*(%))			*X*^2^ = 0.06	0.8
No	77 (15)	63 (14)		
Yes	441 (85)	378 (86)		
Missing	0	1		
Children living at home (*N(*%))			*X*^2^ = 2.9	0.09
No	314 (68)	234 (62)		
Yes	151 (33)	144 (38)		
Missing	53	64		
Type of cancer (N(%))			*X*^2^ = 32.1	< 0.001
Breast	304 (59)	306 (69)		
Digestive	77 (15)	32 (7)		
Skin	25 (5)	9 (2)		
Hematologic	22 (4)	24 (5)		
Gynecologic	17 (3)	15 (3)		
Sarcoma/bone	16 (3)	8 (2)		
Prostate	14 (3)	19 (4)		
Lung	14 (3)	19 (4)		
Other	24 (5)^†^	9 (2)^††^		
Missing	5	1		
Time since diagnosis (in years) (mean ± SD) (range)	2.2 ± 3.4 (0.0–29)	1.2 ± 1.9 (0.0–19)	*t* = 5.6	< 0.001
Missing (*N*)	23	42		
Treatment type (*N*(%))			*X*^2^ = 12.7	0.18
Surgery	98 (19)	72 (16)		
Surgery + radiotherapy	86 (17)	89 (20)		
Surgery +chemotherapy	105 (21)	72 (16)		
Surgery + radiotherapy + chemotherapy	128 (25)	132 (30)		
Surgery + immunotherapy and/or hormonal therapy	8 (2)	14 (3)		
Radiotherapy	7 (1)	7 (2)		
Chemotherapy	49 (10)	30 (7)		
Radiotherapy + chemotherapy	21 (4)	19 (4)		
Immunotherapy and/or hormonal therapy	1 (0)	0 (0)		
Watchful waiting	4 (1)	3 (1)		
Missing	11	4		
Treatment intensity (*N* (%))			*X*^2^ = 1.1	.29
Non-intensive (surgery only, RT only, watchful waiting)	109 (22)	82 (19)		
Intensive	398 (79)	356 (81)		
Missing	11	4		
Treatment intent (*N*(%))			*X*^2^ = 7.1	0.01
Curative	429 (85)	396 (90)		
Palliative	78 (15)	42 (10)		
Missing	11	4		
Treatment phase (*N*(%))			*X*^2^ = 1.0	0.61
Watchful waiting	2 (0.4)	3 (0.7)		
Under active treatment	253 (50.3)	203 (46.7)		
Follow-up	258 (49.3)	229 (52.6)		
Missing	5	7		

^†^10 liver, 9 urologic, 3 head/neck, 1 brain, 1 unspecified

^††^3 liver, 2 urologic, 2 head/neck, 1 brain, 1 unspecified

### DT&PL

DT mean scores were not significantly different between the cohorts nor were percentages scoring under or above the cut-off (Table 2). Compared with C1 respondents, C2 respondents had significantly lower mean scores on problems in the practical (*t* = 2.3; *p* = 0.02; Cohen’s *d* = 0.15), social (*t* = 2.3; *p* = 0.03; Cohen’s *d =* 0.15), and emotional (*t* = 2.9; *p* = 0.004; Cohen’s *d* = 0.19) domains. Mancova’s correcting for differences between cohorts (sex, type of cancer, time since diagnosis, and treatment intent) resulted in comparable findings between cohorts for the practical (*p* = 0.008), social (*p* = 0.038), and emotional (*p* = 0.006) domains. Due to the large overlap between sex and cancer type, the analyses were also performed without the type of cancer; results between groups were comparable: practical *p* = 0.007; social *p* = 0.038; and emotional *p* = 0.005.

**Table 2:** Descriptives on the DT&PL and comparisons between cohorts 1 (pre-implementation) and 2 (post-implementation)

	C1		C2		Test value	*p* value
	Mean (SD)	*N*	Mean (SD)	*N*		
DT	3.8 (2.6)	458	3.5 (2.5)	415	*t* = 1.6	0.1
Cut-off (*N*(%))					*X*^2^ = 2.7	0.1
< 5		272(59)		269 (65)		
≥ 5		186(41)		146 (35)		
Practical	4.6 (7.4)	493	3.5 (6.3)	431	*t* = 2.3	0.02
Social	1.4 (3.7)	501	0.9 (3.2)	432	*t* = 2.3	0.03
Emotional	13.3 (17.3)	485	10.2 (14.4)	424	*t* = 2.9	0.004
Spiritual	1.2 (3.0)	494	0.8 (2.8)	355	*t* = 1.8	0.07
Physical	28.4 (29.9)	471	25.7 (27.9)	419	*t* = 1.4	0.16
Referral wish (*N*(%))					*X*^2^ = 3.3	0.19
No		330 (66)		282 (66)		
Maybe		105 (21)		77 (18)		
Yes		64 (13)		71 (17)		

*N* varies due to missing data

No significant difference was found between the cohorts in responses on the referral wish question (Table 2).

### QoL, anxiety, depression, and patients’ satisfaction with care

No statistically significant differences were found between C1 and C2 on any of these outcome measures (Table 3).

**Table 3:** Descriptives on the EORTC-QLQ-C30, HADS, and Patient Satisfaction Questionnaire-III and comparisons between cohorts 1 (pre-implementation) and 2 (post-implementation)

	C1		C2		*t* test	*p* value
	Mean (SD)	*N*	Mean (SD)	*N*		
EORTC-OLQ-C30						
Global	73.2 (20.1)	508	72.9 (19.9)	437	− .25	0.80
Physical	79.0 (19.0)	501	79.1 (18.8)	432	.08	0.94
Role	71.6 (28.0)	505	73.0 (27.6)	434	.78	0.44
Emotional	82.7 (18.6)	502	82.8 (18.2)	433	.07	0.95
Cognitive	83.3 (20.2)	507	82.8 (20.1)	434	− .44	0.66
Social	83.0 (22.1)	506	84.3 (20.0)	438	.95	0.34
HADS						
Total	8.2 (6.9)	497	8.0 (6.8)	423	.54	0.59
Anxiety	4.7 (3.6)	504	4.5 (3.7)	429	.85	0.40
Depression	3.5 (3.6)	507	3.5 (3.7)	433	.27	0.79
Patient Satisfaction Questionnaire-III						
Total	80.7 (13.4)	503	81.1 (12.8)	436	.55	0.58
Technical quality	78.8 (15.8)	504	78.6 (15.4)	436	− .30	0.76
Interpersonal behavior	83.9 (15.4)	508	84.4 (14.9)	437	.61	0.54
Accessibility	78.6 (14.4)	502	79.4 (13.7)	433	.99	0.32
General satisfaction	81.1 (18.9)	506	80.9 (19.3)	435	− .08	0.94

*N* varies due to missing data

## Discussion

The present comparative two-cohort pre-post implementation sequential study examined the effects on PRO’s of a process of distress and referral need screening (SDRN) implemented in real-world oncology practice. After SDRN had been implemented in clinical practice, cancer patients reported significantly lower symptom presence and severity in the practical, social, and emotional domains of the PL than before SDRN had been part of standard care. These results suggest a beneficial effect. However, no differences were found between the two patient groups in the level of distress, the spiritual and physical domains, and in referral wish, in QoL, anxiety, and depression, or in patients’ satisfaction with care, indicating no beneficial effects of SDRN. Consequently, our results add to the mixed results reported in the literature that vary between a beneficial effect on patients’ well-being, symptom burden, and on quality of care process measures [5–8] and no effect of distress screening on such outcomes [9, 10].

Interestingly, we did find significant differences in the psychosocial domains of the DT&PL, the instrument used for screening and communication in the SDRN process. This result is comparable to three studies with a similar two-cohort pre-post implementation sequential design that also used the screening instrument assessing distress and/or cancer-specific symptoms and concerns as outcome measure [6–8]. A possible explanation is that completing and discussing the results of a screening instrument helped C2 respondents with their psychosocial problems. Talking with a caregiver about a possible referral and receiving extra information about self-management strategies might be enough for some patients to reduce the presence and severity of their distress. However, differences appear to be clinically not very relevant.

Regretfully, no difference was found on the other outcome measurements. It may well be that questionnaires designed to examine group-level differences (e.g., EORTC-QLQ-C30) and non-cancer-related questionnaires (e.g., HADS) measure a different and broader construct than what patients initially experience as the added value of a process of SDRN, namely providing clearer opportunity for discussion of cancer-specific psychosocial problems and the possibility to be referred for further care.

The mixed results in the present study raise the question not only if the study design chosen or the instrument used is the optimal choice to demonstrate an effect of distress screening but also if it is realistic to expect that distress screening in itself can result in improvement in patients’ functioning. As to this last point, we did find that more frequent and fuller exposure to SDRN is positively associated with patients’ satisfaction with distress screening [18]. This would argue for careful assessment of how systematic the SDRN process steps are performed during treatment and follow-up. Distress screening is designed simply to recognize cancer patients’ psychosocial problems and possibly refer them to specialized care. Whether patients who receive a referral actually take up treatment has not yet been researched to date nor has it been researched what the effect is of the treatment that referred patients do undergo. This is unfortunate, as supportive interventions from psychosocial and allied health care professionals have been reported to be effective in terms of QoL, anxiety, and depression and patients’ functioning [21–23] and exactly those correctly identified patients in need may benefit from supportive interventions [24]. Future research should longitudinally study patients who do want a referral separately from those who do not. As not all patients who receive a referral actually follow through with the referral, patients who do take up treatment should be followed separately from those who do not. In addition to researching the effects of distress screening, referral, and take up of services, future studies should also investigate possible reduction in utilization of medical services and associated costs [25].

The current study has the following limitations. First is the design of the study. To examine whether a distress screening process affects outcomes such as patient functioning or referral, a randomized controlled trial may be a more robust type of research. In a clinical situation, randomizing patients is unethical and keeping patients unaware of a difference in (treatment) approach hardly possible. The advantage of our design is the representation of “real-world” everyday clinical practice in which SDRN is implemented. An RCT is an artificially controlled approach to everyday clinical practice. However, our study design may mean that the effects we found cannot be completely attributed to SDRN but could have resulted from other causes or circumstances.

This study was also not a longitudinal study. As mentioned above, future research should follow patients longitudinally to measure effects on the PRO’s of psychosocial and/or allied care interventions to which patients were referred and possibly accepted as a result of the SDRN process.

Second, the response rate was 53% in C1 and 54% in C2, which is comparable to psychosocial studies in oncology [26] but lower than the mean response rate of 70% reported in a recent systematic review [27]. We cannot compare responders to non-responders due to study design. Therefore, we cannot be certain whether respondents are a selection of the total group. There are many reasons why people may decide not to complete a questionnaire, for example not only that they were too distressed but also that they were not distressed at all. This and also other reasons for not participating apply equally to both cohorts. Still this may have affected the representativeness and generalizability. However, this study was conducted in nine hospitals and included patients with varying socio-demographic and illness-related characteristics.

Patients in the cohorts differed in some respects. Compared with C1, more C2 respondents were female, more had breast and fewer digestive cancers, time since diagnosis was shorter, and fewer were treated with a palliative intent. Some of these characteristics have been associated with decreased functioning (female gender, shorter time since diagnosis) [28–30] or were found to be unrelated (treatment intent, breast or digestive cancer) [31]. Given these demographic differences, one would expect to find higher distress in C2 patients. Remarkably, we found slightly lower distress in C2 compared with that in C1, which is promising.

In conclusion, in hospitals that had implemented SDRN, patients report significantly lower presence and severity of problems in the psychosocial (practical, social, and emotional) domains, the domains for which distress screening has been developed and advocated. This is promising and could stimulate care professionals to implement or improve a SDRN process. However, better-designed and more focused studies are needed.
